# Contribution of arbuscular mycorrhizal fungi to the bioavailability of micronutrients (iron and zinc) in millet accessions

**DOI:** 10.3389/fpls.2024.1364469

**Published:** 2024-04-23

**Authors:** Hassna Founoune-Mboup, Bassirou Diallo, Rabiath Féichokpè Raïssa Adigoun, Aboubacry Kane, Abdoulaye Fofana Fall

**Affiliations:** ^1^ National Research Laboratory on Plant Production (LNRPV), Senegalese Agricultural Research Institute (ISRA), Dakar, Senegal; ^2^ Department of Plant Biology, Cheikh Anta Diop University of Dakar, Dakar, Senegal

**Keywords:** bioinoculant, micronutrient deficiency, iron, *Glomus mosseae*, zinc

## Abstract

**Introduction:**

Micronutrient deficiencies, particularly iron (Fe) and zinc (Zn) deficiencies, are prevalent public health issues in developing countries, with children under 5 years old and breastfeeding women being the most affected in Senegal. Agronomic approaches, including soil fertilization and microbial biotechnology, are used to alleviate these deficiencies, yet challenges persist due to poor nutrient availability in staple food crops like pearl millet (*Pennisetum glaucum* L.).

**Methods:**

This study aimed to assess the contribution of one arbuscular mycorrhizal fungal (AMF) strain, *Glomus mosseae*, to the bioavailability of micronutrients (zinc and iron) in pearl millet biomass. Four pearl millet accessions from the National Laboratory for Research on Plant Production (LNRPV) collection were inoculated with *G. mosseae* obtained from the Common Microbiology Laboratory (LCM), with four replications. Gaussian regression tests were employed to analyze the data and determine correlations between AMF concentration and micronutrient bioavailability.

**Results:**

The results indicate that the combination of *Glomus mosseae* inoculation and organic residual products improved growth parameters and micronutrient absorption in pearl millet accessions. Analysis revealed significantly greater iron, zinc, phosphorus, and potassium contents in the foliar biomass of mycorrhizal pearl millet plants compared to non-mycorrhizal plants (control). Inoculation with AMF facilitated micronutrient absorption, particularly iron and zinc, not only in roots but also in aerial parts of the plants.

**Discussion:**

These findings suggest that incorporating AMF and organic residual products in millet cultivation could be a viable strategy for enhancing plant development and increasing iron and zinc contents in varieties. Further research is needed to elucidate the mechanisms underlying AMF-mediated nutrient uptake and optimize their use in agricultural practices.

## Introduction

Iron and zinc deficiencies are two major malnutrition problems in women and children in sub-Saharan Africa. In Senegal, the prevalence of iron and zinc deficiency is estimated to be 66% and 18%, respectively, in children under the age of 5 years. In addition, more than half of the women in the population have a high iron deficiency (Haddad et al., 2015; ANSD/ICF, 2018). These deficiencies are due to low micronutrient food intake (Krebs et al., 2014; Harika et al., 2017). The government of Senegal is investigating how to develop pearl millet (*Pennisetum glaucum* L.) production to improve food security and reduce malnutrition (Vilakati et al., 2016; Govindaraj et al., 2019). Pearl millet is the second most common source of caloric food in this country and the least expensive source of iron and zinc. The average consumption of this cereal is 30.2 kg/person/year in Senegal, of which the average consumption is 53.3 kg/person/year for residents of rural areas and 23.1 kg/person/year for those in urban areas (IPAR, 2018). However, the yield of pearl millet remains low (296 kg/ha), while the potential is 3000 kg/ha (Bamba et al., 2019). The production of this cereal is facing many constraints, such as poor soil fertility management (Diallo et al., 2017), progressive soil salinization, high soil acidification (Dasylva et al., 2019) and erratic rainfall patterns (Faye et al., 2018). Therefore, appropriate and sustainable ways to improve the fertility of soils and increase crop yields are urgently needed to improve nutritional quality. A better alternative would be to use agroecological and environmentally friendly practices based on the joint production of knowledge, combining science and traditional and indigenous knowledge of producers (Food and Agriculture Organization (FAO), 2018). Several approaches have been developed, including the application of organic residual products (ORPs) (Kaboré et al., 2011), the use of nitrogen-fixing green manure (Akhtar et al., 2020), and the use of beneficial rhizospheric microorganisms such as plant growth-promoting rhizobacteria (PGPR) and arbuscular mycorrhizal fungi (AMF) (Diedhiou et al., 2016).

Mycorrhizal symbiosis is a beneficial relationship between most terrestrial plants and AM microorganisms that play an important role in plant hydromineral nutrition (Rodriguez and Redman, 2008). Fungi promote better plant growth and improve nutrient acquisition by forming a large and complex mycelial network in the soil that can absorb phosphorus beyond the depletion zones around the host plant roots (Smith and Read, 2008). AMF improve the uptake of major nutrients such as nitrogen and phosphorus under stress conditions (Jansa et al., 2019; Song et al., 2020), as well as micronutrients such as zinc, iron, and copper (Begum et al., 2019) in the plant. Several studies have demonstrated the positive effect of AMF on pearl millet (Diop et al., 2013; Beggi et al., 2016; Oruru et al., 2018; Mofini et al., 2020). The development of arbuscular mycorrhizal symbioses and the responsiveness of plants depend on the environmental conditions and the specific combinations of plants and fungi (Davison et al., 2015; Diedhiou et al., 2016; Rúa et al., 2016). Exploiting the potential of AMF symbiosis in crop production requires the selection of an appropriate combination of cultivar, fungus, and farming practices.

In addition, ORPs are potential organic fertilizers for agriculture (Kaboré et al., 2011) because their use improves the soil nutrient content (Alburquerque et al., 2012; Mohamed et al., 2018) and, consequently, the yield and nutritional quality of cultivated plants. Nutrients in ORPs can therefore reduce mineral fertilizer inputs and effectively correct soil micronutrient deficiencies (Tambone et al., 2010). The positive effect of combining AMF and ORP on improving plant growth and nutritional quality has been demonstrated in many studies (Abdullahi et al., 2014; Cobb et al., 2018; Pelealu et al., 2019). These studies from different areas have shown that the synergistic effects of soil amendments and AMF reduce agricultural costs while maintaining or/and improving crop yields and quality. It is also important to highlight that the positive response of plants to organic fertilization in combination with AMF differs from the type of organic fertilizer used as well as the AMF strain (Sadiq et al., 2012; Abdullahi et al., 2014; Jiang et al., 2021). Hence, in this context, the objective of this study was to improve pearl millet yield by using AMF biofertilizers and organic residual product combinations.

## Materials and methods

### Experimental setup and treatments

The pot experiment was conducted in the greenhouse facilities of the “Institut Sénégalais de Recherches Agricoles” (ISRA) located in Dakar, Senegal. The experimental design involved the utilization of 288 plastic pots, each filled with 1 kg of soil sourced from the ISRA experimental station in Nioro, situated in southwestern Senegal at coordinates 13°75 N, 15°80 W. Notably, the soil utilized in this study was not subjected to sterilization prior to experimentation.

Four distinct local varieties of pearl millet (GB8735, SL23, SL423, and THIALACK_II) were selected for investigation, all of which were obtained from the collection of the “Laboratoire National de Recherches sur les Productions Végétales” (LNRPV) at ISRA.

### Inoculation and organic residual products

The inoculum utilized in this study was sourced from the “Laboratoire Commun de Microbiologie (LCM; ISRA)” and consisted of spores of *Glomus mossea*, soil with mycelia, and mycorrhized maize roots at a concentration of 100 propagules per gram. Additionally, cow dung and horse manure were chosen as organic residual products (ORPs) due to their ready availability in the target production area. The nutrient contents of these ORPs were analyzed at the “Laboratoire des Moyens Analytiques (LAMA)” of the “Institut de Recherche pour le Développement (IRD), in Bel Air (Dakar-Senegal)” (refer to **Table 1** for nutrient content details).

**Table 1:** Characteristics of the organic residual products used.

Average content	Cow dung	Horse manure
pH	8.20	8.14
C/N	12.29	16.15
Organic matter (kg t^-1^)	66.10	174.99
Dry matter (kg t^-1^)	266.57	721.43
Carbon (kg t^-1^)	30.78	77.18
Total Nitrogen (kg t^-1^)	2.39	4.77
Total Phosphate (kg t^-1^)	0.62	1.11
Potassium (kg t^-1^)	0.82	3.44
Calcium (kg t^-1^)	2.81	8.19
Magnesium (kg t^-1^)	1.45	3.50
Sodium (kg t^-1^)	0.09	0.25
Iron (kg t^-1^)	1.27	4.29
Zinc (kg t^-1^)	0.01	0.03

### Determination of ORP application rates

The application rates of cow dung and horse manure were determined based on their nutrient content (N, P, and K) in conjunction with the nutritional requirements of the pearl millet plants, as outlined in previous studies (Ouendeba and Teme, 2011; Mbaye et al., 2014) and summarized in **Table 2**. Inorganic fertilizers were additionally applied to fulfill the N and K requirements of the crops, as specified in **Table 2**.

**Table 2:** The nutrient content in the ORP and plant requirements.

Parameters	Pearl Millet (y = 2 t ha^-1^)
N, P, K requirement in (kg ha^-1^) of the crop for a yield (y)	N = 60 P = 31.5 K =30
Number of plants ha^-1^	13 333
Cow dung quantity supplied (g)	16.94
Quantity of Urea supplemented (g)	28.08
Quantity of K_2_O supplemented (g)	0
Horse manure quantity supplied (g)	9.46
Quantity of Urea supplemented (g)	26.31
Quantity of K_2_O supplemented (g)	0

### Description of treatment combinations

The treatments encompassed various combinations of AMF inoculation and organic fertilization. The treatments were denoted as follows:

T0: Control (no inoculation or organic fertilization).

T1: *Glomus mossea* inoculation only.

T2: Cow dung application only.

T3: Horse manure application only.

T4: *Glomus mossea* inoculation combined with cow dung application.

T5: Glomus mossea inoculation combined with horse manure application.

### Parameters measured

#### Root mycorrhization

The roots from each pot were harvested and stained using the method described by Phillips and Hayman (1970). The roots were then placed in test tubes, after which KOH (10% (w/v)) solution was added, and the tubes were heated in a Bain-Marie oven at 90°C for 60 minutes. The roots were then rinsed thoroughly to remove the KOH. Trypan blue solution (0.05%) and 0.8% acid acetic solution were added to each test tube for staining at 90°C for 30 minutes (Phillips and Hayman, 1970). The roots were rinsed with distilled water and mounted on microscope slides with glycerol. The stained roots were observed using a compound microscope at 400× magnification. The intensity of mycorrhization was calculated using the following formula Trouvelot et al. (1986):


$$I\%=\frac{\left(95\text{n}5+70\text{n}4+30\text{n}3+5\text{n}2+\text{n}1\right)}{Total\;number\;of\;observed\;fragments}\;$$

where n5, n4, n3, n2, and n1 represent the number of fragments scored as 5, 4, 3, 2, and 1, respectively.

### Plant tissue nutrient contents

The nutrient content in the plant tissue was assessed via LAMA. Shoot samples were oven dried at 60°C for 72 h, after which the dry biomass was recorded. The dried shoots were ground, and the N, P, and K tissue contents were quantified. The pot contents were washed using a 2 mm sieve to isolate the roots kept in ethanol (70%) at 4°C for further evaluation of mycorrhizal root colonization. Roots were stained with 0.05% trypan blue (Phillips and Hayman, 1970), and the percentage of colonization was determined using the method described by (Trouvelot et al., 1986).

Leaf samples were oven dried at 60°C for 2 hours. The dried leaves were ground, and the total phosphorus, potassium, iron, and zinc contents were quantified using microwave plasma atomic emission spectroscopy (4210 MP-AES)) (Li et al., 2013). The leaf chlorophyll content was measured every 7 days from the third week after sowing until harvest using a soil plant analysis development meter (SPAD meter) (SPAD-502 PlusKonica Minolta, Japan). Plant height was measured with a tape every 7 days from the third week after sowing until harvest from the base of the main plant stems to the tip of the longest leaf. The shoot and root samples were oven-dried at 80°C for 72 hours, and the dry biomass was recorded using a TE124S analytical balance (Sartorius). Roots were stained using the method of Phillips and Hayman (1970), and the intensity of mycorrhization was estimated using the method described by Trouvelot et al. (1986).

### Statistical analysis

Descriptive statistics were computed for all the parameters evaluated, and the Shapiro-Wilk normality test was employed to assess data distribution. One-way analysis of variance (ANOVA) was performed for each variety of data that met the normality and homoscedasticity assumptions to test whether there was a significant difference between the different treatments for each of the parameters assessed. The Kruskal–Wallis test was performed for data that did not meet the normality and homoscedasticity assumptions. Multiple comparison tests (Tukey HSD test for ANOVA and Dunn’s test for the nonparametric test at the 0.05 probability level) were also performed to determine the differences among treatments for each variety. The correlation matrix was constructed with Spearman’s coefficient to determine the relationships between the parameters evaluated. Principal component analysis (PCA) followed by hierarchical clustering of the principal components was performed to group the combinations of varieties and treatments based on the evaluated parameters. All graphical representations and statistical analyses were performed using R software version 4.1.2 (R Core Team, 2021).

## Results

### Effects of AMF inoculation and organic fertilization on pearl millet growth

The results showed that the combination of AMF inoculation with organic fertilization significantly increased the growth parameters of pearl millet. At 4 weeks after sowing, no significant differences were observed in terms of plant height or leaf chlorophyll content for any of the pearl millet varieties except for SL23, which presented a significantly greater chlorophyll content in the control T0 treatment than in the T4 and T5 treatments (**Table 3**). Significant differences in plant height among the treatments were observed at 8 weeks after sowing for the SL23 (*p* = 0.0059) and THIALACK_II (*p* = 0.0012) varieties. Eight weeks after sowing, the maximum plant heights for SL23 and THIALACK_II were observed for T2 (67.87 ± 11.28 cm) and T4 (67.55 ± 12.01 cm), respectively. Among the pearl millet varieties, GB8735 exhibited significantly higher chlorophyll content compared to the other varieties in treatments T2 and T4, as shown in **Table 3**. These treatments represent different combinations of AM fungi inoculation and organic residual product application. The chlorophyll content in GB8735 was notably higher under these specific treatments, highlighting the effectiveness of the AM fungi and organic residual products in promoting chlorophyll production in this variety.

**Table 3:** Effect of AMF inoculation and organic fertilization on pearl millet growth parameters.

Pearl millet varieties	Treatments	Plant height at 4 WAS (cm)	Chlorophyll content at 4 WAS	Plant height at 8 WAS (cm)	Chlorophyll content at 8 WAS	Shoot biomass (g)	Root biomass (g)
GB8735	T0	43.54 ± 8.27ab	17.65 ± 7.05a	58.15 ± 5.69a	13.38 ± 2.93b	0.95 ± 0.23ab	0.41 ± 0.12a
	T1	33.24 ± 15.8b	17.5 ± 7.63a	53.48 ± 12.54a	13.82 ± 2.07b	0.92 ± 0.76ab	0.6 ± 0.45a
	T2	51.82 ± 5.21a	13.67 ± 3.65a	67.63 ± 12.98a	19.1 ± 4.15a	1.4 ± 0.18ab	0.63 ± 0.24a
	T3	49.37 ± 11.45ab	15.3 ± 2.91a	56.43 ± 7.58a	15.67 ± 0.65ab	1.09 ± 0.34ab	0.62 ± 0.32a
	T4	54 ± 5.66a	13.4 ± 2.51a	62.03 ± 5.87a	17.27 ± 1.24ab	1.56 ± 0.14a	0.45 ± 0.19a
	T5	36.25 ± 13.98b	16.4 ± 4.01a	54.07 ± 8.39a	16.17 ± 2.2ab	0.82 ± 0.37b	0.7 ± 0.27a
SL23	T0	35 ± 6.81a	24.02 ± 4.6a	51.17 ± 8.56c	15.77 ± 3.09a	0.98 ± 0.45a	0.88 ± 0.56a
	T1	32.68 ± 13.02a	21.93 ± 4.04ab	53.75 ± 8.1bc	14.78 ± 3.9a	0.99 ± 0.57a	0.92 ± 0.51a
	T2	44.88 ± 10.45a	18.15 ± 3.74ab	67.87 ± 11.28a	16.87 ± 1.63a	1.65 ± 0.62a	0.79 ± 0.39a
	T3	40.5 ± 6.4a	16.62 ± 5.93ab	59.84 ± 1.29abc	14.28 ± 1.9a	1.52 ± 0.58a	0.88 ± 0.52a
	T4	42.2 ± 3.62a	15.25 ± 1.87b	65.7 ± 7.15ab	17.28 ± 2.82a	1.2 ± 0.41a	0.6 ± 0.34a
	T5	39.2 ± 6.65a	14.85 ± 4.25b	56.9 ± 6.84abc	15.37 ± 1.95a	1.03 ± 0.29a	0.4 ± 0.22a
SL423	T0	35.68 ± 13.78a	18.14 ± 3.73a	54 ± 11.47ab	14.98 ± 3.44a	0.9 ± 0.63ab	0.93 ± 0.43a
	T1	30.73 ± 8.23a	15.33 ± 5.35a	40.72 ± 7.88b	15.85 ± 2.81a	0.43 ± 0.15b	0.66 ± 0.15a
	T2	40.52 ± 5.89a	15.98 ± 2.42a	59.28 ± 6.58a	18.37 ± 2.5a	1.15 ± 0.27a	0.53 ± 0.11a
	T3	44.42 ± 7.26a	18.03 ± 4.2a	61.08 ± 9.48a	15.12 ± 2.87a	1.27 ± 0.4a	0.53 ± 0.19a
	T4	42.3 ± 5.44a	16.5 ± 3.18a	59.05 ± 10.78a	18.28 ± 3.09a	1.13 ± 0.13a	0.6 ± 0.13a
	T5	36.78 ± 7.88a	16.53 ± 3.69a	48.92 ± 7.96ab	16.15 ± 2.05a	0.85 ± 0.32ab	0.57 ± 0.22a
THIALACK_II	T0	29.93 ± 14.41a	16.43 ± 2.89a	42.92 ± 11.12c	15.83 ± 4.25a	0.62 ± 0.31b	0.67 ± 0.28a
	T1	32.83 ± 7.84a	18.77 ± 3.88a	46.38 ± 8.25bc	14.22 ± 2.04a	0.61 ± 0.28b	0.43 ± 0.1a
	T2	41 ± 15.19a	14.25 ± 2.98a	62.62 ± 9.02ab	15.55 ± 2.25a	1.37 ± 0.5a	0.5 ± 0.25a
	T3	39.87 ± 11.72a	15.15 ± 1.06a	58.7 ± 13.39abc	13.95 ± 1.7a	1.1 ± 0.56ab	0.6 ± 0.47a
	T4	46.25 ± 10.13a	16.82 ± 3.31a	67.55 ± 12.01a	17.2 ± 1.95a	1.77 ± 0.87a	0.63 ± 0.33a
	T5	43.37 ± 5.59a	15.42 ± 1.92a	60.03 ± 5.12abc	15.5 ± 2.58a	1.09 ± 0.09ab	0.41 ± 0.13a

For each variety and within a column, values with the same letter do not differ significantly (p< 0.05). WAS, Week After Sowing.

Moreover, there were no significant differences in shoot biomass among the treatments for SL23 (*p* = 0.1689). For the varieties GB8735 (*p* = 0.0108) and SL423 (*p* = 0.0035), the maximum shoot biomass was recorded for T4 and T3, respectively. There was no significant difference between the T4 and T3 treatments compared to the control T0 treatment. Only THIALACK_2 plants treated with T4 or T2 had greater shoot biomass (*p* = 0.0032) (1.77 ± 0.87 g and 1.37 ± 0.5 g, respectively) than did those in the control T0 treatment (0.62 ± 0.31 g). For the root biomass, no significant differences among the treatments were observed for any of the varieties (**Table 3**).

### Effects of AMF inoculation and organic fertilization on the mycorrhization of pearl millet

**Figure 1:**
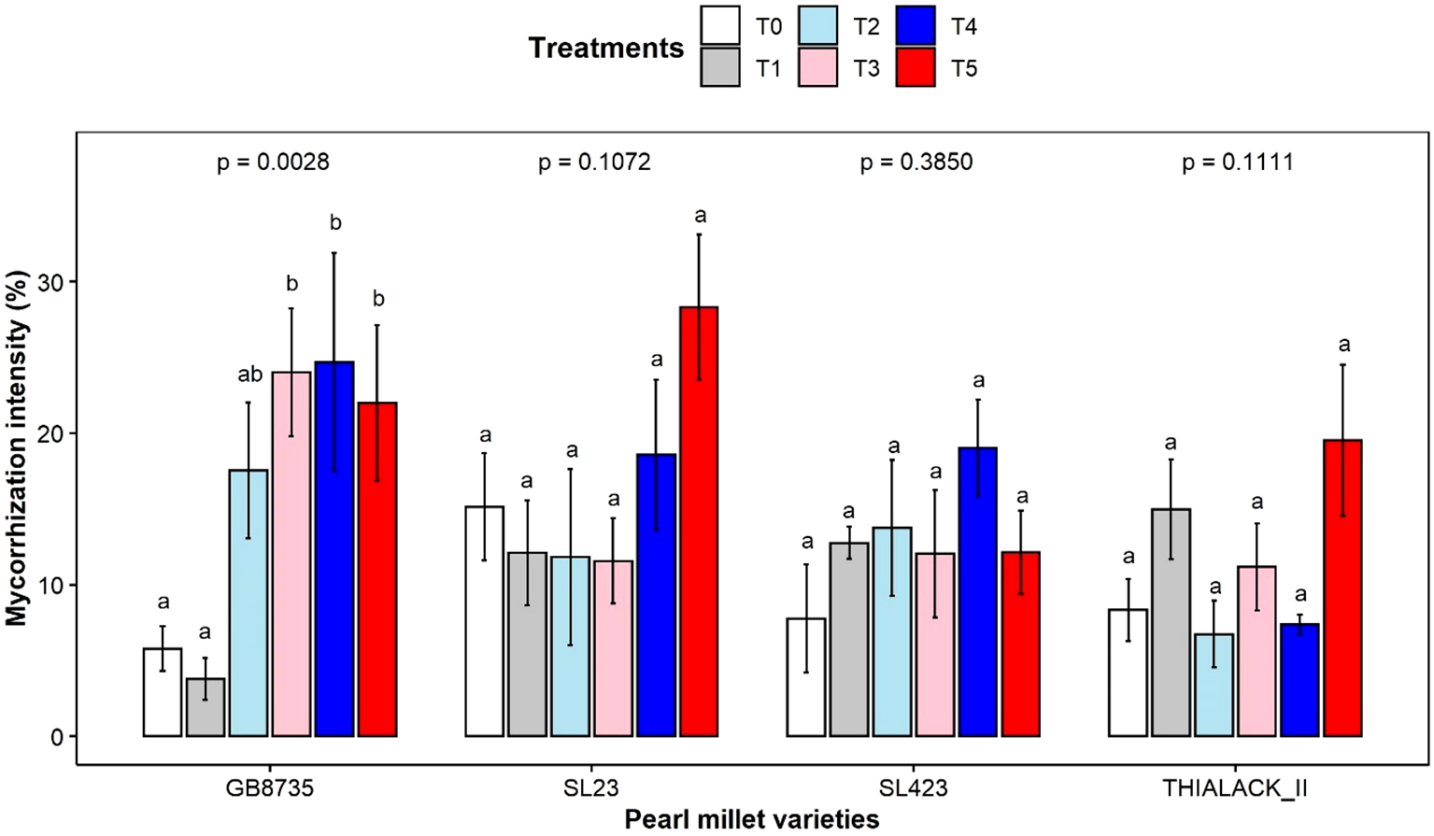
Effect of *G. mosseae* inoculation and organic fertilization on the intensity of mycorrhization in pearl millet varieties. T0, control; T1, *G. mosseae*; T2, cow dung; T3, horse manure; T4, *G. mosseae* + cow dung; and T5, *G. mosseae* + horse manure. The bars represent the standard deviations. For each variety, values with the same letter do not significantly differ (p< 0.05).

No significant differences in the intensity of mycorrhization were observed for the SL23, SL423, or THIALACK_2 varieties (**Figure 1**). In contrast, compared with control T0, GB8735 had a significantly greater percentage (*p* = 0.0028) of mycorrhized T4 (24.69 ± 17.63%), T3 (24.02 ± 10.3%) and T5 (21.99 ± 12.59%) (5.77 ± 3.62%) (**Figure 1**). For SL23, the highest intensity of mycorrhization was observed for T5 (28.32 ± 10.70%).

### Correlation analysis of the parameters evaluated for pearl millet

Spearman’s correlation analysis revealed that there was a strong positive correlation between plant height and shoot biomass (r = 0.79; *p*< 0.001), while the correlation between chlorophyll content and root biomass was weak and negative (r = -0.18; *p* = 0.032). The root biomass weakly correlated with shoot biomass (r = 0.25; *p* = 0.003) and mycorrhization intensity (r = -0.17; *p* = 0.039).

### Principal component analysis of pearl millet

**Figure 2:**
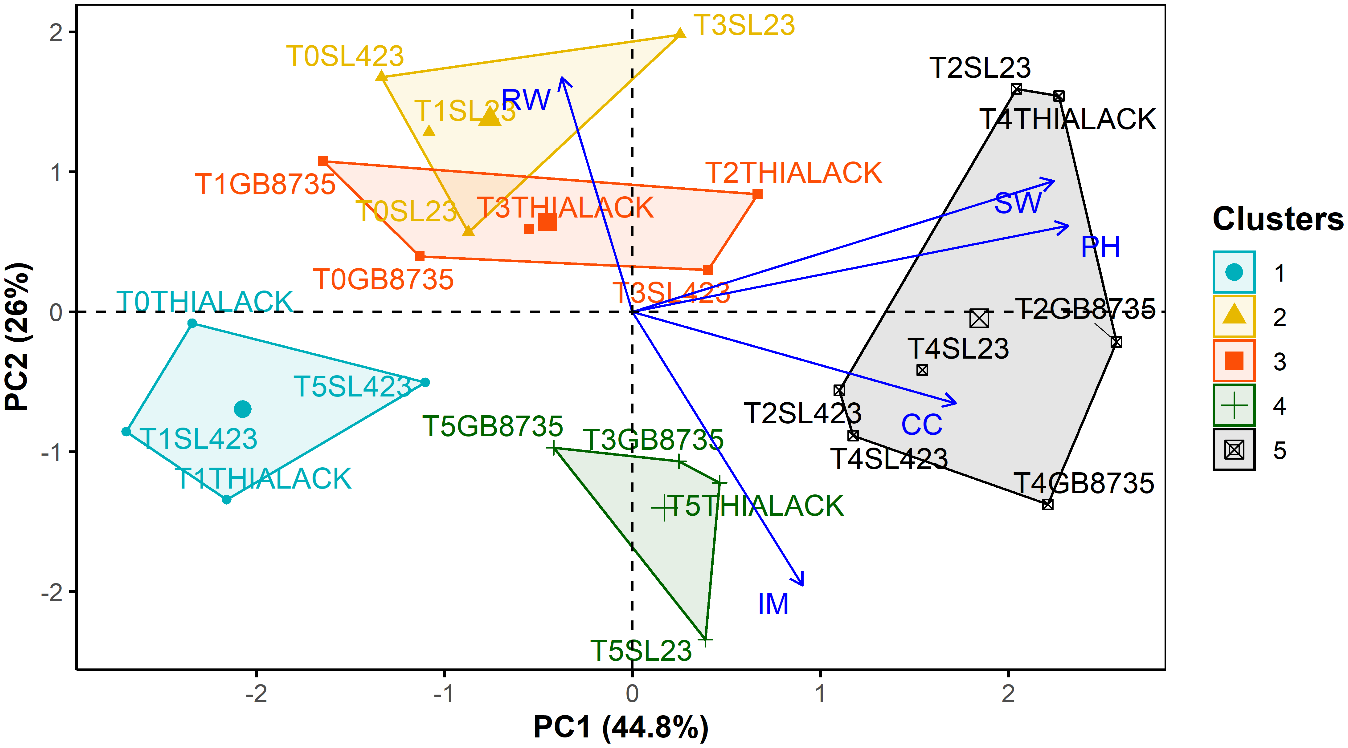
Principal component analysis biplot of the combinations of treatments and pearl millet varieties based on plant height (PH), chlorophyll content (CC), shoot biomass (SW), root biomass (RW), and mycorrhization intensity (IM). Treatments: T0 (control), T1.

**Figure 3:**
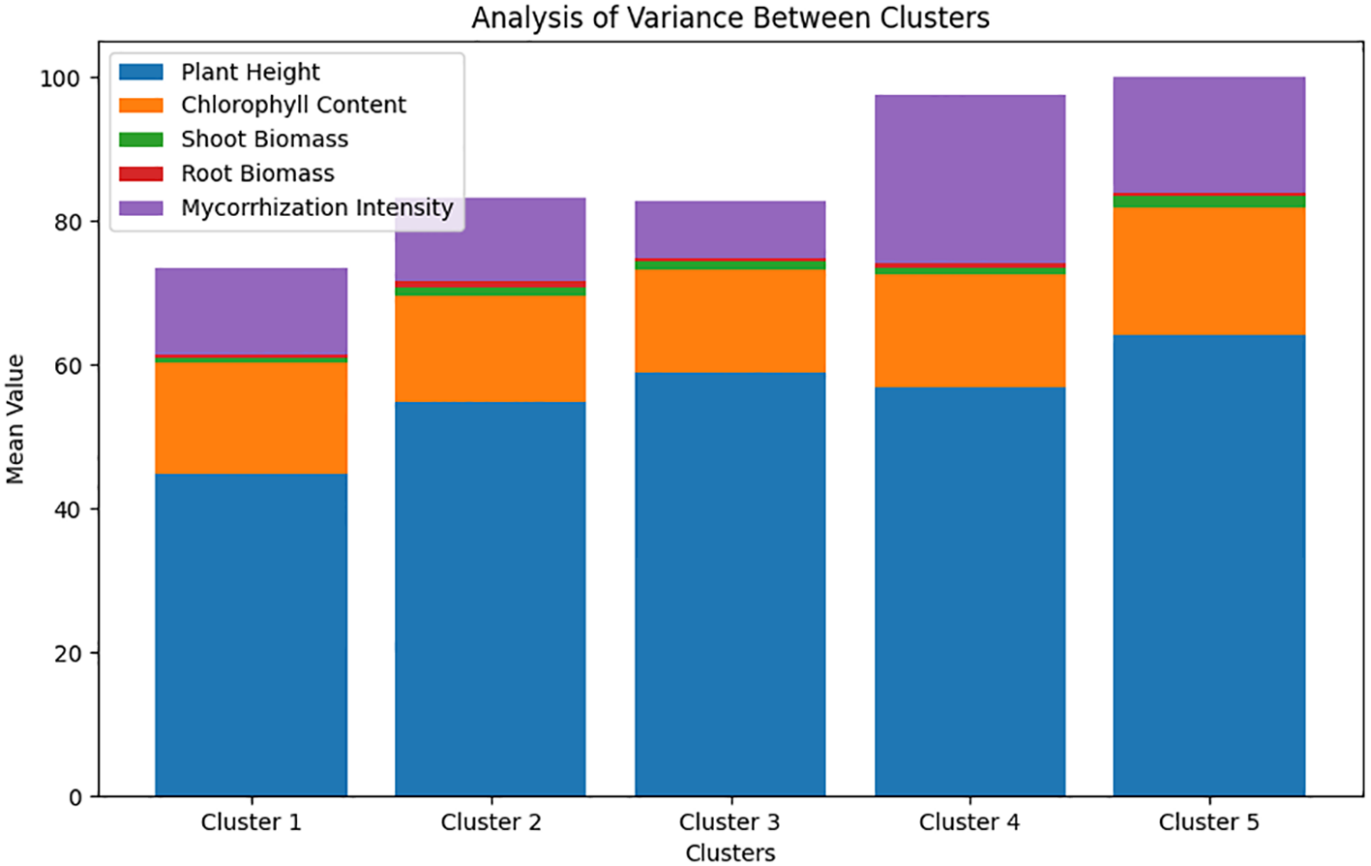
Comparative analysis of pearl millet varieties: understanding variability in growth and physiological parameters across clusters.

Principal component analysis (PCA) revealed that the first two components explained 70.8% of the total variation (**Figure 2**). Plant height, chlorophyll content, and shoot biomass were significantly and positively correlated with the first principal component. The second principal component was opposed and significantly correlated with root biomass and mycorrhization intensity (**Figure 2**). Furthermore, the hierarchical clustering of principal components grouped the combinations of varieties and treatments into five clusters. Analysis of variance (**Figure 3**) revealed significant differences among the clusters and revealed that the treatments in cluster 5 (T2SL23, T2GB8735, T4THIALACK, T4SL23, T4GB8735, T2SL423, and T4SL423) were those associated with significantly high growth parameters and high mycorrhization intensity. This cluster contained mostly varieties that received cow dung fertilizer treatment. The plants in the Cluster 4 treatment groups (T5SL23, T3GB8735, T5GB8735, and T5THIALACK) also exhibited significantly increased mycorrhization intensity and were characterized by the variety that received mainly horse manure.

### Leaf mineral content and association with growth and mycorrhizal parameters for the pearl millet varieties SL23 and SL423

**Figure 4:**
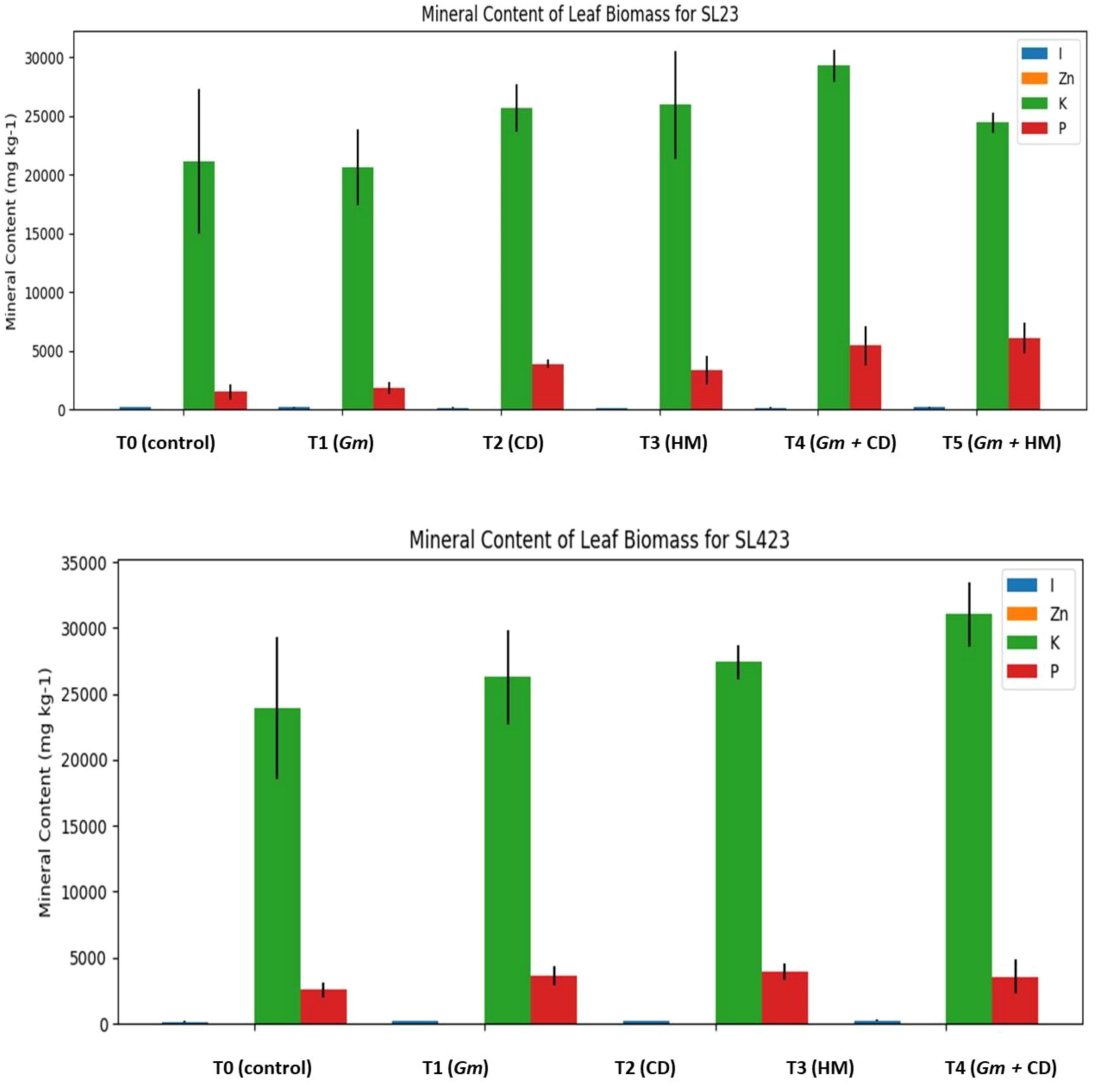
Leaf mineral content of different millet varieties under various treatments. Gm, *Glomus mosseae*; CD, cow dung; HM, horse manure; I, Iron; Zn, Zinc; K, Potassium; P, Phosphorus; mean ± standard deviation.

The assessment of mineral content in the leaf biomass of SL23 and SL423 pearl millet varieties under various treatments offers valuable insights into nutrient uptake dynamics. For SL23, treatment T5 (*Glomus mosseae* + horse manure) demonstrated the highest zinc and phosphorus levels, while treatment T4 (*Glomus mosseae* + cow dung) exhibited elevated iron content. Conversely, in SL423, treatment T4 (*Glomus mosseae* + cow dung) displayed the highest iron levels, with treatment T5 (*Glomus mosseae* + horse manure) showcasing superior zinc and phosphorus accumulation. Notably, treatment T2 (cow dung) in SL423 recorded the highest potassium content (**Figure 4**).

**Figure 5:**
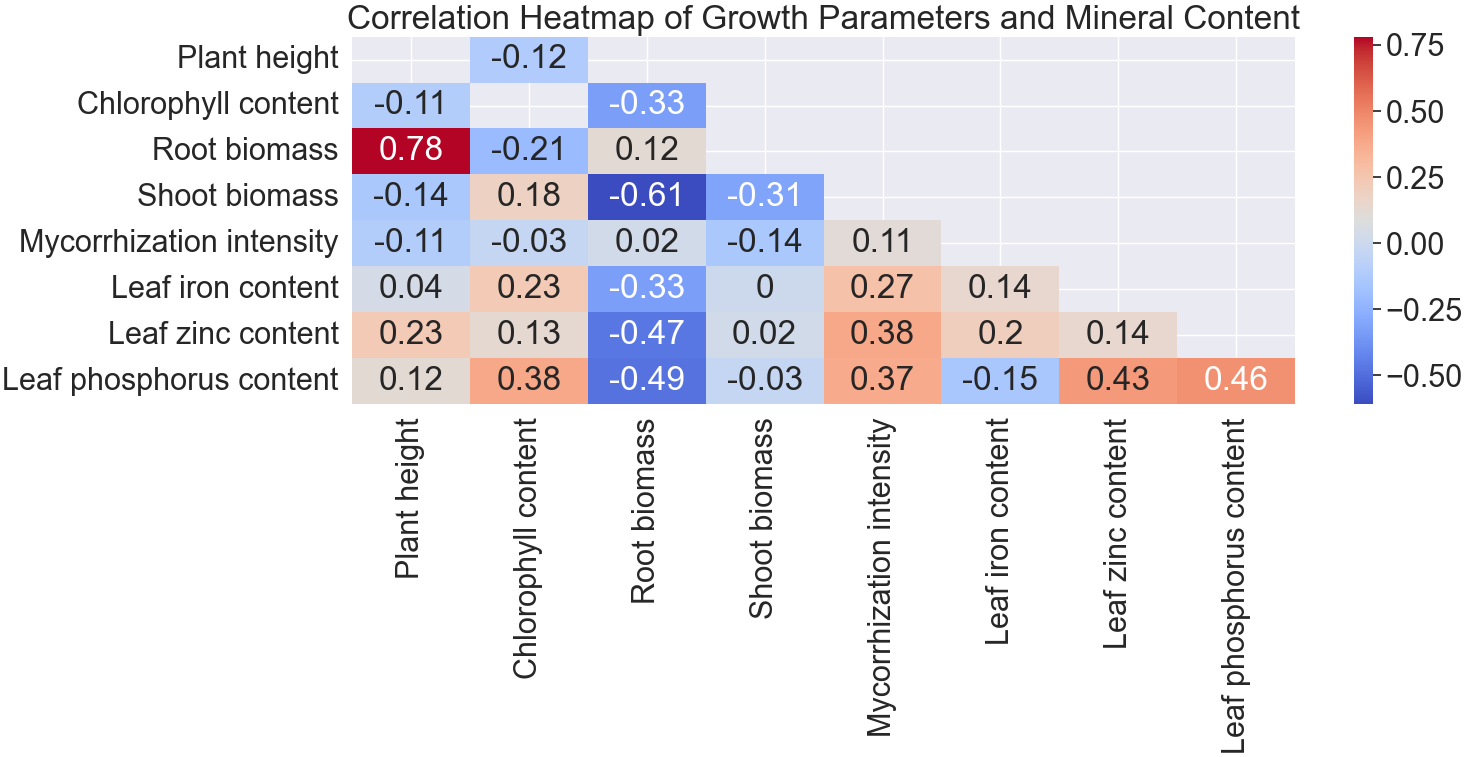
Correlation heatmap of growth parameters and mineral content.

The correlation heatmap offers valuable insights into the relationships among various growth parameters, mycorrhization intensity, and mineral content in pearl millet plants. The heatmap vividly illustrates the strength and direction of these correlations, allowing for a comprehensive understanding of the intricate interplay between different factors influencing plant development (**Figure 5**). For instance, strong positive correlations are observed between shoot biomass and plant height (0.78***), indicating that taller plants tend to exhibit greater above-ground biomass. Conversely, a negative correlation is noted between shoot biomass and root biomass (-0.61***), suggesting a trade-off in resource allocation between above-ground and below-ground structures. Additionally, mycorrhization intensity shows positive correlations with shoot biomass (0.18) and negative correlations with root biomass (-0.31), underscoring the potential influence of mycorrhizal fungi on plant growth dynamics. Furthermore, leaf iron content (LI) and leaf zinc content (LZn) demonstrate positive associations with shoot biomass and mycorrhization intensity, highlighting the significance of these minerals in plant development and symbiotic interactions. Overall, the correlation heatmap provides a comprehensive visual representation of the complex relationships governing pearl millet growth and nutrient acquisition, facilitating targeted strategies for enhancing crop productivity and nutritional quality.

## Discussion

All the control and inoculated pearl millet varieties were mycorrhized since the soil was not sterilized. Mycorrhization intensity was low (ranging from 3.7% to 15.14%), which corroborates the results of Mofini et al. (2020), who showed that cultivated and wild millet species contract mycorrhizal symbiosis but with low mycorrhization rates. The soil collected from Nioro used in this study contained a significant number of spores of the genera *Glomus* and *Gigaspora*, indicating the presence of an indigenous AMF community in the soil used, which explains the mycorrhization of noninoculated plants. The lack of difference between the treatments that were inoculated and those that were not inoculated for most of the growth parameters could be explained by the fact that the native AMF strains were able to provide the plant with nutrients capable of photosynthesis in the absence of the inoculation of the selected AMF strain (Duponnois et al., 2001). The AMF strain *Glomus mosseae* was therefore not effective in the presence of the existing indigenous AMF strains for root colonization of the pearl millet varieties since a significant effect was not obtained on root mycorrhization. The lack of a significant effect of mycorrhization on growth in the GB8735, SL23, and THIALACK_II varieties shows that pearl millet is not dependent on AMF, although these fungi can stimulate its growth (Plenchette et al., 2000; Mofini et al., 2020).

Organic fertilization affected the indigenous AMF microbial community in the presence or absence of the selected AMF strain. Cow dung, alone or in combination with the *Glomus mosseae* strain, enhanced plant growth, while horse manure improved the mycorrhization of the roots of all the varieties. These results confirm those of Sadiq et al. (2012), who recorded greater growth and yield of pearl millet treated with cow dung than poultry manure. Abdullahi et al. (2014), on the other hand, showed that poultry manure promoted pearl millet growth more than cow manure did. These discrepancies could be explained by the differences in the mineral composition of the different types of organic fertilizers used. In this study, horse manure was much richer in nutrients (especially N, P, and K) than was cow dung, and the highest mycorrhization intensity was observed with horse manure. The additional mineral fertilizer (urea) applied in the cow dung treatment could also explain the difference in growth between the pearl millet varieties and the horse manure treatment because the nutrients provided by the mineral fertilizers are immediately available to the plants. This difference in results between the two types of ORP could also be due to their mineralization over time (Morvan et al., 2006). The effects of AMF inoculation combined with organic fertilization were variety dependent because the results showed different responses for each variety.

The results also revealed an increase in the mineral content of the leaves due to AMF inoculation combined with organic fertilization. Cow dung and AMF promoted an increase in the zinc and phosphorus contents of SL23 leaves. On the other hand, horse manure with AMF increased the potassium content of the SL23 leaves and the iron content of the SL423 leaves. In addition, the mycorrhization intensity was positively correlated with the phosphorus and potassium contents of the leaves. This means that the more AMF colonize plant roots, the greater the P and K leaf tissue content. These results confirm the work of Abdullahi et al. (2014), who showed the positive impact of AMF inoculation combined with organic fertilization on nitrogen, phosphorus, and potassium uptake in pearl millet plants. According to Coccina et al. (2019), AMF play a significant role in the uptake of Zn by cereals, especially wheat and barley, depending on the plant species and available soil Zn. The important role of AMF in improving the uptake of inorganic phosphorus (Pi), nitrogen, potassium, and magnesium in shoots has also been demonstrated in maize (Zhao et al., 2015; Garcés-Ruiz et al., 2017).

Several studies have shown the positive effect of AMF on the bioavailability of nutrients, particularly phosphorus, potassium, zinc, and iron, in plants (Marschner and Dell, 1994; Clark and Zeto, 2000; Ortas and Bilgili, 2022). However, although the results showed an improvement in nutrient uptake by plants, the discrepancies observed can be justified by the type of organic manure applied and the presence of indigenous fungi, which, in the presence of the inoculated fungus, did not provide a microbial balance favorable for the optimal absorption of some nutrients by the plant. Indeed, the contribution of AMF to nutrient acquisition depends on fungal-specific effects on the activity of the plant pathway and on the efficiency with which both partners interact and exchange nutrients across the mycorrhizal interface (Bücking et al., 2012). Jiang et al. (2021) argued that organic fertilizer increases AMF biomass and is less harmful to AMF richness than mineral fertilizer is. The same authors highlighted that AMF generally responded positively to organic fertilizer when AMF and host plants had strong mutualistic symbioses, such as in phosphorus-deficient soil, drought and semidown areas; at low latitudes; and at testing sites that contained two or more plant species or included legumes (Jiang et al., 2021).

## Conclusion

This research study evaluated the effect of the combination of organic fertilization and mycorrhizal inoculation on the growth of four pearl millet varieties. The results revealed that *Glomus mosseae* alone did not stimulate the growth of pearl millet. Organic fertilization alone or in combination with *Glomus mosseae* promoted optimal growth; high mycorrhization; and zinc, iron, potassium, and phosphorus uptake in all the pearl millet varieties. Cow dung alone or combined with *Glomus mosseae* significantly improved plant growth, while horse manure improved root mycorrhization in all the varieties. However, a field study in which *Glomus mosseae* would be tested in combination with organic fertilizer is recommended for evaluating the effects of *Glomus mosseae* on pearl millet and other cereals and legumes because pot trials might have affected the results of the study.

## Data availability statement

The original contributions presented in the study are included in the article/supplementary files, further inquiries can be directed to the corresponding author/s.

## Author contributions

HF: Conceptualization, Funding acquisition, Methodology, Project administration, Resources, Supervision, Validation, Visualization, Writing – review & editing. BD: Conceptualization, Methodology, Supervision, Writing – review & editing. RA: Conceptualization, Data curation, Investigation, Methodology, Writing – original draft, Writing – review & editing. AK: Conceptualization, Methodology, Supervision, Writing – review & editing. AFF: Conceptualization, Formal analysis, Methodology, Validation, Writing – original draft, Writing – review & editing.
